# Use of an Integrated Knowledge Translation Approach to Develop an Electronic Patient-Reported Outcome System for Cancer Rehabilitation: Tutorial

**DOI:** 10.2196/74123

**Published:** 2025-11-24

**Authors:** Christian Lopez, Sarah E Neil-Sztramko, Kristin L Campbell, David M Langelier, Tran Truong, Yuliya Gavrylyuk, Pia Nyakairu, Laura Parente, Audrey Durand, Jackie L Bender, Gillian Strudwick, Rupali Bhati, Jonathan Greenland, Tony Reiman, Jennifer M Jones

**Affiliations:** 1 Department of Supportive Care, Princess Margaret Cancer Centre, 610 University Avenue, Toronto, ON, M5G 2C4, Canada, 1 416 581 8620; 2 Institute of Medical Science, University of Toronto, Toronto, ON, Canada; 3 Faculty of Health Sciences, McMaster University, Hamilton, ON, Canada; 4 National Collaborating Centre for Methods and Tools, McMaster University, Hamilton, ON, Canada; 5 Department of Physical Therapy, University of British Columbia, Vancouver, BC, Canada; 6 Department of Clinical Neurosciences, University of Calgary, Calgary, AB, Canada; 7 Techna Institute, University Health Network, Toronto, ON, Canada; 8 Healthcare Human Factors, University Health Network, Toronto, ON, Canada; 9 Department of Computer Science and Software Engineering, Université Laval, Québec City, QC, Canada; 10 Canada-CIFAR AI Chair, Mila - Quebec Artificial Intelligence Institute, Québec City, QC, Canada; 11 Dalla Lana School of Public Health, University of Toronto, Toronto, ON, Canada; 12 Institute of Health Policy, Management and Evaluation, University of Toronto, Toronto, ON, Canada; 13 Dr H Bliss Murphy Cancer Centre, NL Health Services, St John’s, NL, Canada; 14 Faculty of Medicine, Memorial University of Newfoundland, St John’s, NL, Canada; 15 Department of Biological Sciences and Integrated Health Initiative, University of New Brunswick, Saint John, NB, Canada; 16 Department of Oncology, Saint John Regional Hospital, Saint John, NB, Canada; 17 Department of Medicine, Dalhousie University, Saint John, NB, Canada

**Keywords:** integrated knowledge translation, electronic patient-reported outcomes, cancer, supportive care, symptom management, rehabilitation, implementation science, digital health

## Abstract

Electronic prospective surveillance models (ePSMs) have the potential to improve the management of cancer-related impairments by systematically screening patients using electronic patient-reported outcomes during and after treatment, and linking them to tailored self-management resources and rehabilitation programs. However, their successful implementation into routine care requires careful consideration of patient and provider needs and must align with clinical workflows, which may vary across settings and require adaptation to the local context. The aim of this paper is to describe the development of REACH, a web-based ePSM designed to remotely screen for physical cancer–related impairments and direct patients to rehabilitation resources based on need. The development of REACH followed an integrated knowledge translation (iKT) approach, engaging key knowledge users including patients, clinicians, administrators, and information technology specialists. The development process involved collaboration across 5 working groups. The system content and logic group selected the impairments to be screened, measures used, frequency of screening, and resources recommended based on results of a survey with oncology providers and researchers, patient feedback, a literature review, and an environmental scan. The machine learning group explored predictive modeling approaches to optimize the assessment frequency using retrospective patient data. The implementation group identified features from existing systems that could be built to promote assessment completion and integration into clinical workflows through a scoping review, interviews with clinic staff, and focus groups with patients. The design group conducted co-design workshops and usability testing with patients to iteratively refine the interface and develop a prototype. Finally, the software development group converted the prototype to a web-based application and conducted privacy and security assessments and quality assurance. The integration of key knowledge users through an iKT approach played a critical role in determining the design and functionality of REACH. REACH allows patients to remotely complete assessments tailored to their cancer type and treatment status on any electronic device. The system generates automated advice based on the assessment responses, including links to educational resources for self-management, suggestions for community programs to register for, and recommendations to contact their oncology team for further assessment and possible referral to rehabilitation services. These recommended resources are stored in the patient’s personalized library, organized by type and severity of cancer-related impairments reported, and are updated following each new electronic patient-reported outcomes assessment completed. Additional key system features include a patient-driven and structured process for managing high impairment scores, usability enhancements to improve navigation, and safeguards to ensure data security. The development of REACH demonstrates how an iKT approach can be used to design an ePSM that is user-friendly, clinically relevant, and aligned with implementation considerations. The system has been implemented at 4 Canadian cancer centers, and its implementation is being evaluated to inform future refinements.

## Introduction

People living with and beyond cancer are at risk of experiencing treatment-related adverse effects that can lead to impairment and disability [1]. It is estimated that 37% of individuals affected by cancer report significant challenges with basic activities of daily living and about 55% report challenges with instrumental activities of daily living along the cancer pathway (eg, diagnosis, adjuvant therapy, and follow-up surveillance) [2]. However, treatment-related adverse effects often go underreported and undetected [3,4], leading to the low uptake of cancer rehabilitation services [5,6].

An electronic prospective surveillance model (ePSM) is an evidence-based digital solution that aims to enable the proactive identification of impairments to promote early self-management, through access to education, as well as facilitate timely referrals to programs and rehabilitation services [7,8]. An ePSM uses electronic patient-reported outcomes (ePROs) to systematically screen and identify rehabilitation needs throughout cancer treatment and follow-up surveillance and provides patients with tailored self-management advice and alerts to clinicians to support the management of identified impairments [7,8]. Studies evaluating ePSMs in cancer care have demonstrated the clinical use of these systems [9], improvements in important patient outcomes such as symptom management and quality of life [10,11], and improvements in health service outcomes such as reductions in emergency department presentations [12]. However, there are noteworthy challenges to their development and implementation, including the need to develop a system that meets the needs of a heterogeneous patient population (eg, cancer types, treatment options, and types of impairments) that can be integrated into diverse clinical settings (eg, availability of rehabilitation resources, variability in workflows, current use of digital solutions, and the use of different electronic medical record systems) [13-15].

An integrated knowledge translation (iKT) approach can help address key development and implementation challenges of ePSMs. iKT involves the partnership between researchers and knowledge users throughout the research and implementation process [16]. In the context of an ePSM, an iKT approach ensures that key knowledge users (eg, patients, health care providers, administrators) play an active role in shaping the system’s design and implementation. This step is critical as it may promote local uptake of an ePSM through a sense of increased ownership by key knowledge users. Furthermore, engaging knowledge users early and throughout the process helps to align an innovation with their needs, values, and workflows, which may enhance an innovation’s clinical use, adoption, and sustainability [17].

Despite the growing use of ePSMs, there remains a need for detailed descriptions of their development to provide insights into the processes and decision-making involved, which can help guide others looking to design and implement similar systems. This paper describes the iKT approach taken to develop an ePSM called REACH prior to its implementation within 4 centers in Canada and 4 disease sites (breast, colorectal, head and neck, and lymphoma). These populations were selected due to their high prevalence and high potential for impact given the documented symptom burden within each disease site. The aim was to ensure the REACH system was tailored to these clinical settings and populations to facilitate its subsequent implementation into routine care.

## Project Team and Governance Structure

The Canadian Cancer Rehabilitation (CanRehab) team comprises a multidisciplinary group of researchers, clinicians, and patients with cancer across Canada. CanRehab operates within a team-based organizational structure that includes a steering committee (ie, study principal investigators, implementation science lead, and training program lead), a patient and family advisory committee (PFAC), and an external advisory committee. The PFAC comprised 13 representatives across Canada, and the external advisory committee comprised 3 international experts in cancer rehabilitation.

## REACH System Development Plan

### Overview

**Figure 1.**
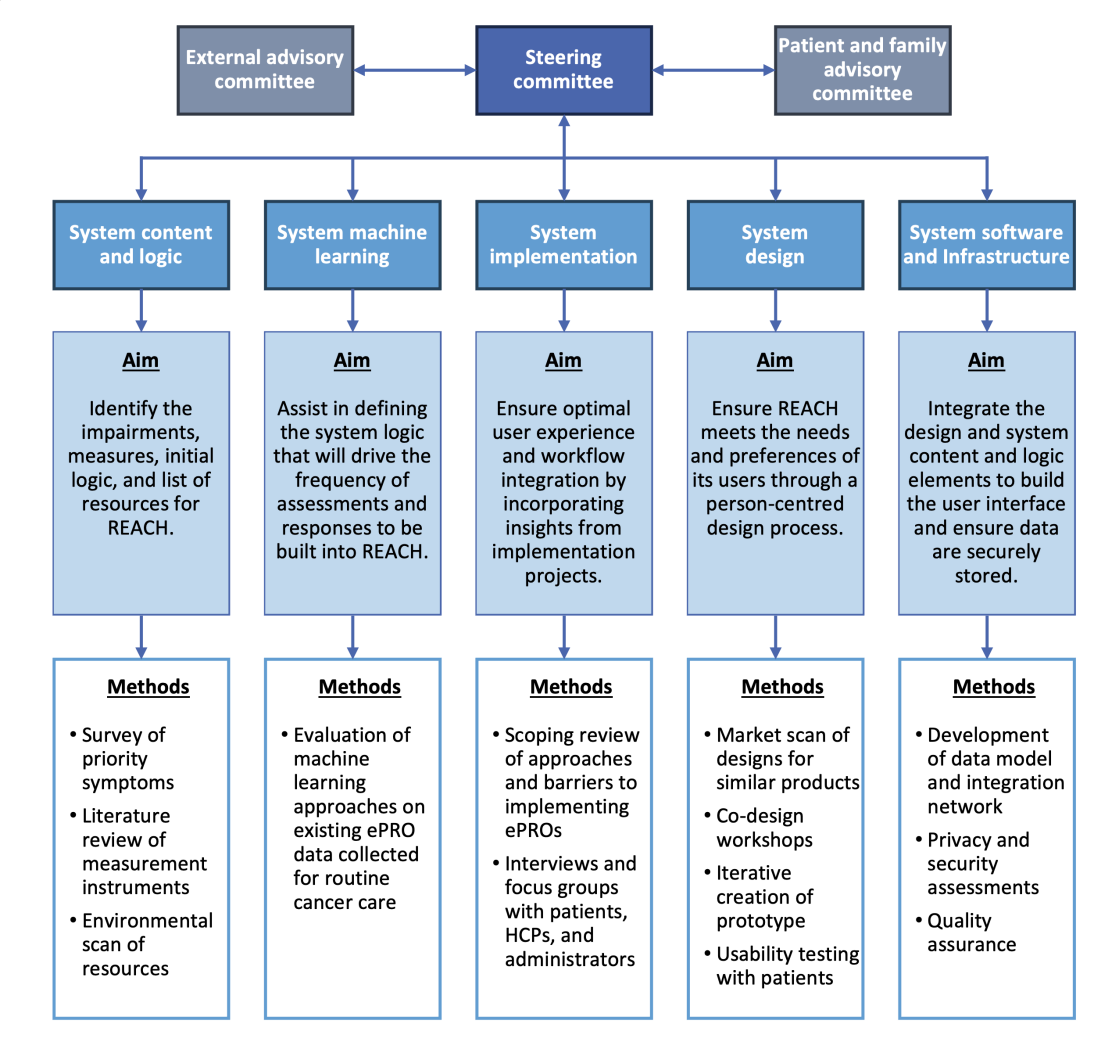
Canadian Cancer Rehabilitation (CanRehab) team organizational structure and process for the development of the REACH system. ePRO: electronic patient-reported outcome; HCP: health care professional.

To ensure local needs were considered in the development of the REACH system, we used an iKT approach. Throughout the development of REACH, we engaged patients through the PFAC, as well as oncology health care providers, administrators, and information technology staff within the settings where REACH will be implemented. The development of REACH also involved the collaboration between 5 working groups, each reporting to the CanRehab steering committee and contributing specialized expertise. These working groups focused on: (1) system content and logic; (2) system machine learning (ML); (3) system implementation; (4) system design; and (5) system software and infrastructure. Each group’s responsibilities, methods, and deliverables are described in detail in the following sections and summarized in Figure 1.

### System Content and Logic

The system content and logic working group was led by team members with expertise in cancer survivorship and physical medicine and rehabilitation (JMJ, KLC, and DML). The development of the initial logic and content of REACH included the completion of three main system components: (1) impairments to screen for each disease site; (2) measurement instruments, cut-offs, and frequency of assessments; and (3) self-management and rehabilitation resources offered to patients.

To determine the impairments that would be monitored for each disease site, the working group conducted a web-based survey. Members of the CanRehab team who were invited to complete the survey included oncologists from all disciplines (eg, medical, radiation, hematology, and surgical oncology) and disease sites that were the focus of the initial implementation of REACH (breast, colorectal, head and neck, and lymphoma), as well as researchers with expertise in patient-reported outcomes, cancer rehabilitation and survivorship, and cancer education. The survey was delivered via SurveyMonkey [18], and all responses were anonymous. Survey participants were provided with 5 lists of approximately 10 impairments (1 list for each of the 4 disease sites and 1 general list). Participants were asked to rank these impairments based on their perceived importance, relevance, and feasibility for assessment and surveillance. Each list had an option to add additional impairments. This activity was issued a formal waiver exempting it from the requirement for research ethics board approval at the University Health Network, as it was determined not to constitute research as described by the Tri-Council Policy Statement [19].

A total of 29 surveys were completed by members within the CanRehab team. To select the final list of impairments for each disease site, this working group synthesized the survey responses and aimed to select the top 5 ranked impairments. Further consideration was given to the availability of resources to offer patients to manage a specific impairment. The final list of impairments selected for each disease site is displayed in Table 1. Briefly, each cancer type includes an assessment for falls and balance, fatigue, pain, and activities of daily living, in addition to impairments specific to each site. Examples include screening for dysphagia and trismus (head and neck cancer), lymphedema (breast cancer), and sexual function (breast and colorectal cancer).

**Table 1.** Impairments and measures selected for REACH.

Impairment	Cancer type	Measures adapted
Fatigue	Breast, colorectal, head and neck, lymphoma	FSI^a^
Pain	Breast, colorectal, head and neck, lymphoma	ESAS^b^ pain and BPI^c^
Activities of daily living	Breast, colorectal, head and neck, lymphoma	PRFS^d^ and FSQ^e^
Falls and balance	Breast, colorectal, head and neck, lymphoma	STEADI^f^
Return to work	Breast, colorectal, head and neck	None
Shoulder and neck dysfunction	Breast, head, and neck	QuickDASH^g^, Neck Dissection Impairment Index
Sexual dysfunction	Breast, colorectal	BSSC-M and F^h^
Lymphedema	Breast	Lymphedema Symptom Report
Dysphagia	Head and neck	4QT^j^
Trismus	Head and neck	EORTC QLQ^i^-H&N35
Speech	Head and neck	UW^k^ Head and Neck Questionnaire
Xerostomia	Head and neck	EORTC QLQ-H&N35

a FSI: Fatigue Symptom Inventory.

b ESAS: Edmonton Symptom Assessment System.

c BPI: Brief Pain Inventory.

d PRFS: patient-reported functional status.

e FSQ: Functional Status Questionnaire.

f STEADI: Stopping Elderly Accident, Deaths, and Injuries.

g QuickDASH: The Disabilities of the Arm, Shoulder, and Hand Score.

h BSSC-M and F: Brief Sexual Symptom Checklist Male and Female.

i EORTC QLQ-H&N35: European Organization for Research and Treatment of Cancer Quality of Life Questionnaire Head and Neck Module.

j 4QT: 4-­Point Questionnaire Test.

k UW: University of Washington.

Next, the system content and logic working group conducted a literature review of measurement instruments for the impairments selected. The working group aimed to use the findings from the literature review to select patient outcome measures for the impairments selected that had been validated and had clear cut-offs. In cases where a validated measure was not identified (ie, screening for difficulties with return to work), the system content and logic working group developed a brief question about the impairment. The proposed questions were presented to the CanRehab external advisory committee, steering committee, and the PFAC, and the questions were refined based on feedback from these groups. Notable feedback included adding a question for each impairment, where if a patient breached the threshold for being referred to a program or service, the system would ask the patient if they had already received a referral and were already being seen by a health care provider for that impairment. The final list of measurement instruments is displayed in Table 1.

The system content and logic working group then conducted site-specific and national environmental scans to identify all self-management and rehabilitation resources (eg, patient education resources, hospital- and community-based programs, and rehabilitation professionals) that may be included as resource options in the system. These were categorized into three types: (1) education, (2) programs, and (3) referrals. “Education” included websites, videos, and online pamphlets that provided information to enable patients to self-manage impairments. “Programs” included online and in-person classes, workshops, and programs delivered by trained professionals in the community. “Referrals” included specialized services associated with or delivered in the cancer center. These resources were mapped to each geographical site, cancer type, and impairment where appropriate. All links to resources (websites, videos, programs, and clinics) were validated by a member of the CanRehab team. Furthermore, all community- and hospital-based programs were contacted to inquire about referral processes (eg, physician referral or self-referral) and patient eligibility criteria to ensure the recommended resources to patients on the system were appropriate.

The environmental scan of resources resulted in a total of 98 resources that were included in the REACH system. Most of the resources were categorized as education (61/98, 62%) compared to programs (16/98, 16%), and referrals (21/98, 21%). Of the 61 (62%) education resources, most were websites (29/61, 48%) and links to online pamphlets (22/61, 36%), followed by videos (8/61, 13%) and e-modules (3/61, 5%). Of the 16 (16%) programs, over half can be delivered either in-person or virtually to patients receiving care at a specific center (9/16, 56%), with the remaining programs being delivered virtually to all patients across all 4 centers (7/16, 44%). Of the 21 services within the referral category, services included outpatient physiotherapy (4/21, 19%), occupational therapy (1/21, 5%), speech-language pathology (4/21, 19%), dietetics (3/21, 14%), lymphedema programs and specialized staff (5/21, 24%), pain management clinics (2/21, 10%), and outpatient rehabilitation programs offering a variety of these services with a multidisciplinary team (2/21, 10%).

**Figure 2.**
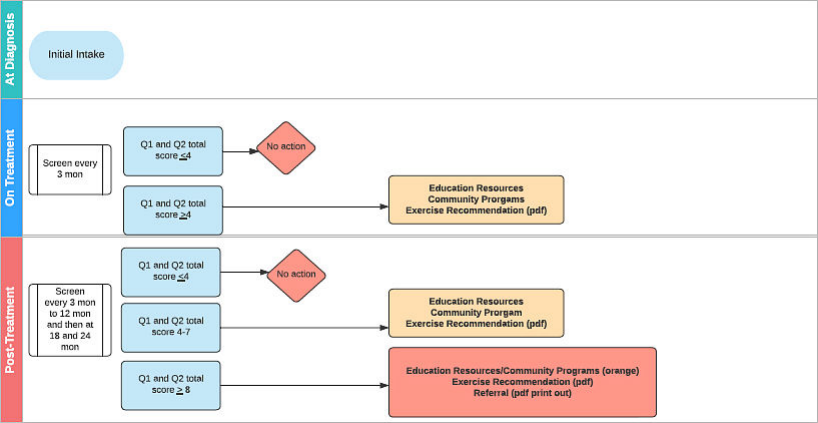
System logic for fatigue. mon: months.

Finally, the system content working group held internal meetings dedicated to each impairment being monitored by REACH. These meetings aimed to establish the algorithms and pathways for each impairment, which were developed using Lucidchart (Lucid Software Inc). These pathways were then presented to the steering committee and the PFAC for their feedback and refinement. A figure of the final logic for each impairment selected (ie, the frequency of assessment during and after treatment, and the type of resource provided based on the patient’s response and clinical characteristics) was created to facilitate the translation of the logic to the REACH system during system software development (see Figure 2 for example for fatigue).

### System ML

This working group was led by a team member with expertise in ML for health-related applications (AD). The purpose of this working group was to evaluate the potential use of ML for predicting impairment levels that would inform the need for a follow-up assessment (or not), thereby assisting the system content and logic working group in defining the system logic for REACH (ie, tailoring the assessment frequency based on a patient’s previous assessment and level of impairment, as well as cut-offs for impairments screened). To achieve this goal, the working group conducted analyses using ML approaches on existing ePRO data collected from one of the centers where REACH would be implemented. The data analyzed were from patients with breast, colorectal, head and neck, and lymphoma collected as part of distress screening at the Princess Margaret Cancer Center (N=36,574 patients with >1 assessment). The analysis focused on 2 impairments, namely pain and fatigue, and extracted the distribution of records over impairment levels (0-10, with 0=lowest symptoms). Full details on the methods and results of this work are reported elsewhere [20]. The ML pipeline relied on light gradient boosting machine [21], an interpretable and efficient classifier based on decision trees, combined with the synthetic minority oversampling technique for handling data imbalance [22]. The working group simulated the training and use phases of the ML pipeline by splitting the data into 2 parts. The working group used 5-fold cross-validation for model selection during training. In evaluation, the ML pipeline was used to predict all impairment level records appearing in the deployment (test) set, and its performance was quantified using the mean average error per class (impairment level) and overall using the weighted mean average error. In a first evaluation setting, the working group assumed that the previous level of impairment would always be available for predicting the next one (as usually performed in ML evaluations). The working group then conducted another evaluation setting (closer to reality) where the ML pipeline would only have access to the previous level of impairment if the patient had been previously selected for a follow-up assessment (ie, based on the predictions of the previous impairment levels).

This evaluation revealed that it would not be appropriate to rely on an ML model to determine the frequency of follow-up assessments based on a patient’s previous level of impairment reported on the REACH system. Specifically, we found that the amount of available data decreased as the impairment level increased, leading to greater prediction errors at higher impairment levels. This imbalance in the data significantly impacted the ML model’s ability to learn and accurately predict across all levels of impairment. In addition, our evaluations suggested that if the frequency of follow-up assessments depends on an ML model, there is a risk that prediction errors would start accumulating over time and prevent the observation of signals that the ML model would require for correcting its future predictions (which are based on the current levels). This was supported by a Shapley Additive Explanations analysis of the ML model, which revealed that the prediction for the current impairment level is mostly based on the previous observed level and the gradient between the 2 previous observed levels. Finally, we observed that using an ML model for deciding when to ask patients to complete a follow-up assessment rapidly translates into a decrease in performance, even if the observation is set to a low value. Given these challenges, we determined that an ML-driven approach to tailoring assessment frequency would not be appropriate for REACH at this point. Further investigations would be required for a safe integration of this technology.

### System Implementation

The system implementation working group was led by individuals with training and expertise in implementation science (CJL, SNS, and JMJ) with additional support from individuals with expertise in implementing digital health interventions within clinical care practices (GS and JB). The design of the implementation plan for the REACH system was conducted in parallel to its development. The working group led a scoping review of the approach to implementing ePSM systems in cancer care and a qualitative study with patients, oncology health care providers, and clinic leadership to identify barriers and facilitators prior to implementing REACH at each of the 4 implementation settings. Details of the methods and results of both studies have been published [13,23]. While the primary aims of these studies were to inform the development of the implementation plan for REACH, the findings from both studies acted as an additional point of consideration during discussions regarding the system’s features to improve user experience and facilitate the integration with clinical workflows.

**Figure 3.**
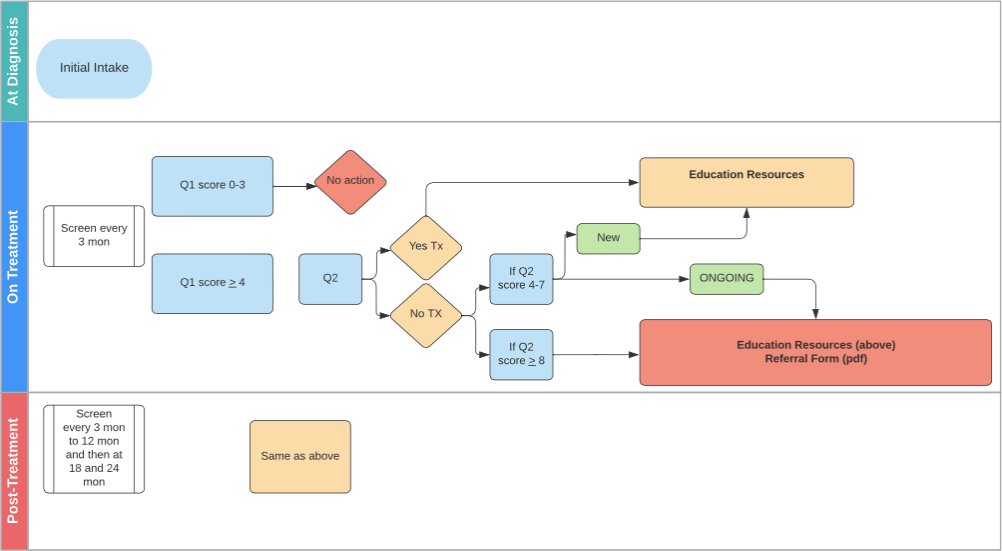
System logic for pain. mon: months; Tx: treatment.

Findings from the scoping review helped support the system content and logic group’s work on defining the initial frequency of assessments on REACH. The scoping review found that most systems did not have a defined assessment schedule for patients and, of those that did, the frequency of assessments and duration of surveillance varied widely. For instance, the frequency of assessments ranged from daily to every 3 months, and the duration of surveillance ranged from one month to 5 years after the completion of treatment. Furthermore, this depended on many factors including where patients were along the cancer pathway, the impairments being screened, and the goal of the assessment (eg, whether the assessment was conducted as part of a clinical visit). These results were integrated into discussions during the internal meetings dedicated to each selected impairment to be monitored by REACH, as well as presentations to the steering committee and PFAC. The team decided on initial frequencies of 2‐3 months during treatment and 3‐6 months after treatment, depending on the impairment being screened (see Figure 3 for example for pain). Several considerations led to this decision, including the system’s purpose of identifying persistent cancer-related impairments rather than acute toxicities, and the importance of minimizing patient burden, particularly during treatment, when patients face competing demands such as intensive treatment schedules and frequent clinic visits. The scoping review also synthesized common features of ePSM systems and found that many systems used various methods to display a patient’s ePRO scores longitudinally (eg, graphs and reports). This was also recommended by patients interviewed in our qualitative study. As such, we developed a feature in the REACH system that allows a patient to view their responses from the most recent assessment. Patients can access this at any time but are also presented with the option to view their previous assessment scores if they log into REACH before their next scheduled assessment.

**Figure 4.**
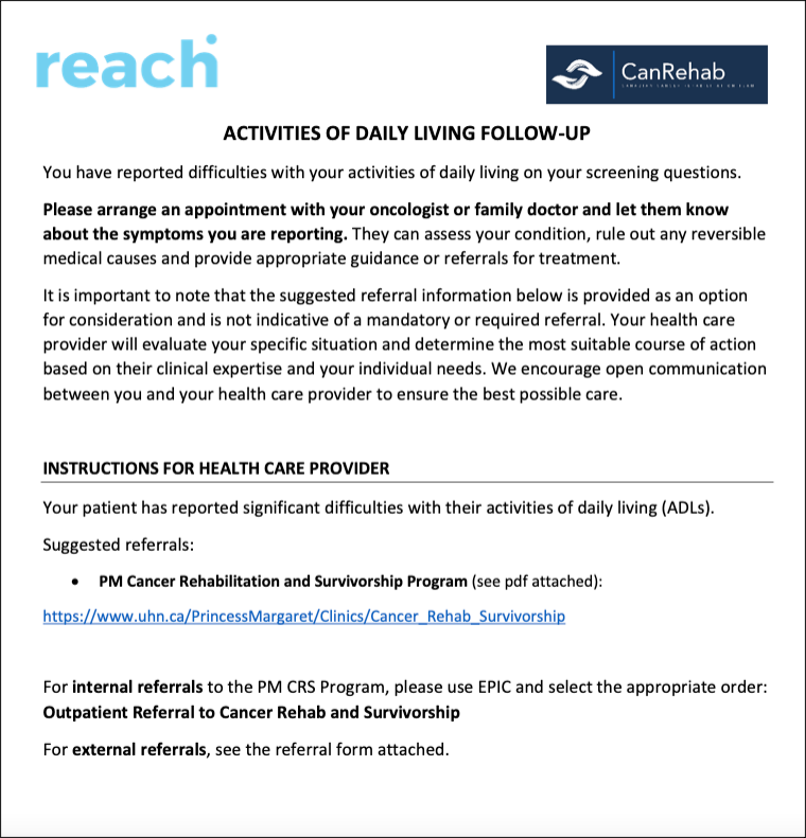
Print out for patients recommended by REACH to follow up with their physician. CRS: Cancer Rehabilitation and Survivorship; mon: months; PM: Princess Margaret Cancer Centre; TX/Tx: treatment.

Findings from the scoping review and the qualitative study also informed the development of a process to manage high scores reported by patients and facilitate referrals to rehabilitation services. Some systems in the scoping review included a method of alerting patients that they should contact their clinical team for further screening, and in certain contexts, that their scores were not being monitored by a provider in real-time. Systems also typically included alerts to the clinical team for patients who breached specific thresholds that require attention, as well as recommended clinical actions and suggested referrals to manage identified impairments. In our qualitative study, interviews with health care providers revealed that certain rehabilitation services that are in the REACH library may require a referral from a physician; however, providers also noted that it would not be feasible for the clinical team to receive alerts related to the REACH system. Therefore, we developed a process on REACH where patients who (1) breach specific clinical thresholds for various impairments assessed and (2) are not currently receiving care for the identified impairment are recommended to schedule a visit with their family physician or oncologist for further screening. Patients are also provided with a printout to bring to their appointment which includes a brief background of the assessment and suggested referrals for the physician to consider (see Figure 4 for example for activities of daily living).

### System Design

The system design working group was led by individuals from the Healthcare Human Factors team (HHF) at the University Health Network. HHF team members included the Director of Design and Strategy (JR) and designers (PN and LP), who supervised and administered a human-centered design approach. The first phase (immerse and understand) involved conducting a market scan for analogous products in other diseases and industries to review successful schematics for providing tailored online recommendations to users. This scan served as a source of inspiration for the end-product, enabling its design to ultimately be both engaging and relevant. A document immersion was also conducted to situate the product within the context of the cancer journey and align it with the overarching system goals. In the second phase (concept design), a unique set of product concepts was generated and brought to 2 virtual co-design workshops with the PFAC to elicit feedback on how to best deliver REACH. During these 3-hour workshops, facilitators from the HHF team led participants through a series of activities to define the value proposition for REACH, identify the rehabilitation resources they relied on throughout the cancer pathway, validate design concepts, and explore how they would come to life in the next phase.

Next, the HHF team began the third phase (design and test) in which they iteratively created the interface and experience of using the system, incorporating feedback along the way. The needs and journeys of the people that the solution aims to serve were captured through a series of user stories that enabled designers to create REACH’s information architecture. From there, a set of wireframes was produced, structured around key patient workflows and later converted into a refined prototype, ready for feedback from end users. In the final step of the third phase, the HHF team conducted usability testing with 9 members of the PFAC. Goals of this usability testing included assessing the user-friendliness of the product and gaining insight into whether the features successfully met the user needs previously identified. Prior to each session, participants were provided with a brief introduction to the testing environment and test materials. During the testing session, participants were asked to perform representative tasks with the application, while test administrators monitored the proceedings for any usability issues that may be indicative of user-safety or usability problems.

**Figure 5.**
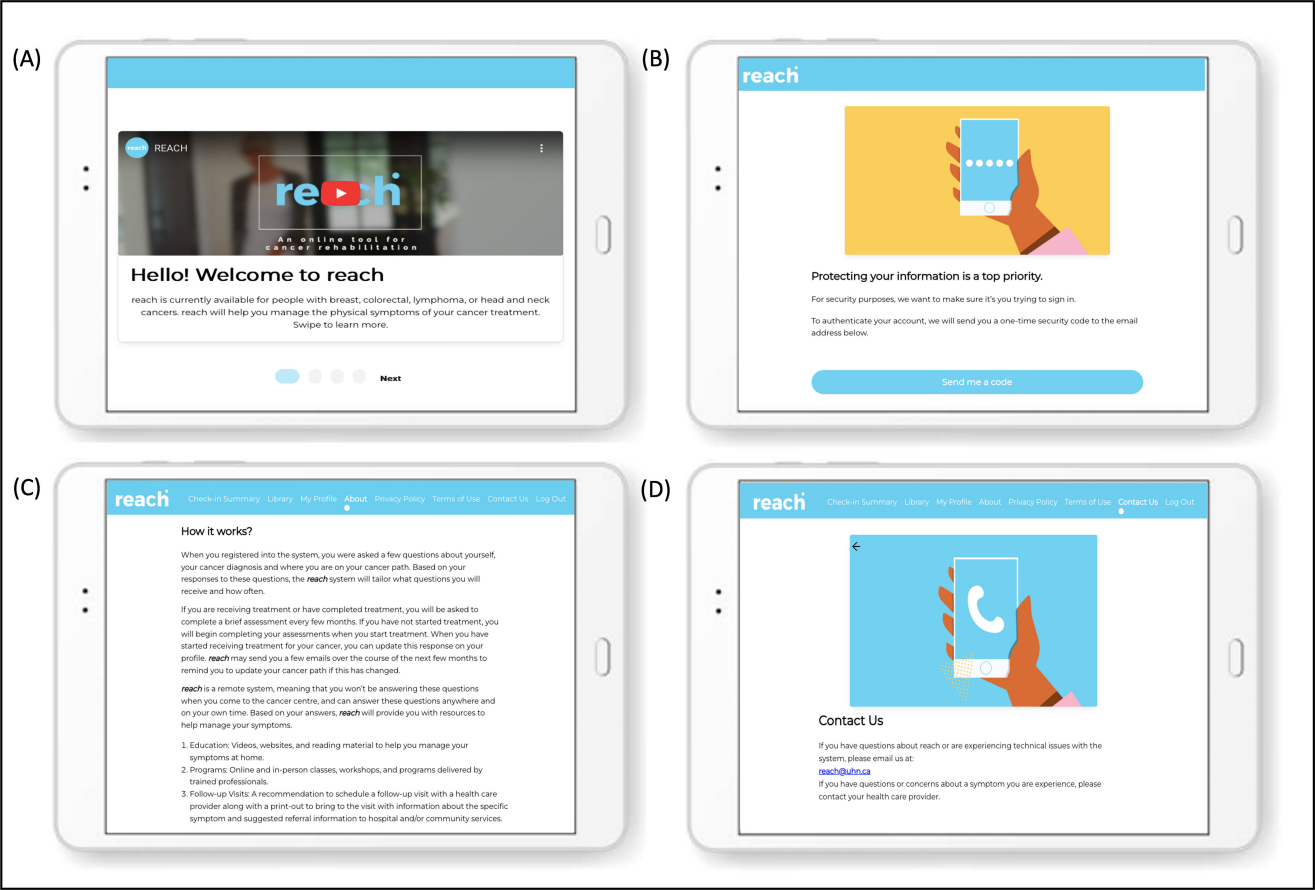
Example screens of key design elements for REACH. (A) Onboarding page outlining the system’s purpose and target population. (B) Multifactor authentication screen for secure login. (C) “About REACH” page providing details on assessments and types of resources offered in the library. (D) Contact information for support with technical issues.

The HHF team then created the final designs of the REACH system, aiming to deliver a user-friendly, visually engaging, and easy-to-navigate platform for patients. Key design elements include an onboarding page that contains brief and accessible information on the purpose of REACH and the steps required to create a user profile (Figure 5). A video demonstration of the process for creating a user profile on the system is provided in Multimedia Appendix 1. Upon logging into the system, patients are provided with a screen displaying the date of their next assessment and a list of resources that were recommended to them during their previous assessment. During an assessment, patients are presented with all the questions for a given impairment on a single page, allowing them to easily navigate between different impairments. Questions that use a scale are accompanied by definitions for each end point (eg, 0=no pain; 10=worst possible pain) to ensure clarity in responses. The resource library is organized by impairment (eg, fatigue, lymphedema, and pain) and resource type (education, programs, and follow-ups), enabling patients to easily locate resources that best match their needs and preferences. In addition, patients have access to various pages, including a “Contact Us” section that provides an email address for inquiries related to REACH or technical issues (Figure 5). A video demonstration of the assessment process and resource library navigation is provided in Multimedia Appendix 2.

### System Software and Infrastructure

Following completion of the final designs, the design and software and infrastructure teams worked closely together to convert the prototype into a web-based application. The system software and infrastructure working group was led by individuals from the Techna team at the University Health Network. The Techna project manager (TT) and a senior analyst (YG) supervised and managed the development work with additional support from a system architect, software developer, and quality assurance specialist. This working group followed a structured software development life cycle process. The team worked together to build a robust and scalable data model, design the integration network, and finalize technical specifications. Furthermore, the system software and infrastructure working group developed the user interface according to the designs developed by the design team, set up the application on a secure web server, and performed quality assurance prior to production. Finally, the team worked with the University Health Network’s privacy, security, and legal teams to conduct a medical device assessment and develop the system’s Terms of Use and privacy policy and embed this into the REACH platform.

Collectively, this work resulted in the development of a secure login process, secure storage of user data, embedded algorithms to support tailored assessments and recommended resources for patients, automated email notifications to patients (registration, password reset, and assessment reminders), and the generation of system logs to support quality improvements. To set up an account on REACH, patients use their own email and password, and multifactor authentication was implemented to support secure user access (Figure 5). Password resets are managed by the system, where patients may submit a forgot password form using their email address. The system stores a unique temporary token for that patient and sends an email to the patient with a link that contains the specific token. Notably, the REACH system consists of a lightweight progressive web application with centralized data storage in a secure data center at the University Health Network. As such, REACH can be accessed on any electronic device and only uses browser local storage to determine if the patient has been prompted to install REACH and if the patient has previously visited the site. No personal health information or identifiable data is stored on the patient’s device. The data are encrypted at rest and in transit. Passwords are salted and hashed using SHA256. The operating system that is used for REACH is Linux/Red Hat EL7 (application) and Oracle Linux v7.9 (database). The application programming interface of REACH has logging and auditing capabilities.

## Discussion

### Overview of the REACH ePSM System

There is a need to develop ePSM systems that meet the needs and preferences of patients, consider the fit with clinical settings, and address potential implementation barriers. The successful implementation of these systems has the potential to address the low reporting and detection of cancer-related impairments during and after treatment and facilitate patient uptake of self-management and rehabilitation resources. This paper describes the process of using an iKT approach to develop an ePSM system, called REACH.

REACH is a web-based application that systematically screens for common cancer-related impairments and provides patients with tailored rehabilitation resources based on their reported needs. Registered patients create a user profile by providing their sex, age, cancer type, treatment status, and institution receiving care. The system then personalizes the type and schedule of assessments accordingly. Patients receive automated email notifications when assessments are due and are provided with tailored resources based on their responses. These resources, including links to education resources for self-management, community programs to register for, and recommendations to follow-up with their physician with suggested referrals, are stored in each patient’s personalized resource library, which remains accessible for viewing and use at any time.

REACH was intentionally designed as a patient-driven system in which patients self-enroll and complete assessments independently, outside of clinical encounters. As such, integration with the electronic medical record was not prioritized, since the tool is intended primarily to support self-management rather than to inform real-time clinical decision-making. In addition, the 4 implementation sites used different electronic medical record systems, making technical integration complex and resource-intensive. To facilitate uptake while maintaining feasibility, REACH was instead designed to align with existing clinical workflows by being introduced through standard patient education processes. These include presurgical classes, systemic therapy and radiation therapy teaching sessions, and inclusion in educational packages distributed to patients. Strategies to promote awareness and uptake, such as handouts, posters, videos, and point-of-care staff introductions, were tailored to each site and are detailed in a separate publication describing the REACH implementation strategies [24].

The integration of diverse knowledge users through an iKT approach played a critical role in shaping the final design and functionality of the REACH system. Several key system features were directly influenced by input from patients, clinicians, administrators, and information technology staff, highlighting the importance of ongoing engagement in system development. For instance, in response to health care provider concerns about feasibility, a structured process was developed for managing high impairment scores, recommending patients to bring a printout to their physician instead of generating automated alerts to clinicians. Furthermore, to ensure resources are directed to those in need, a follow-up question was added for each impairment (ie, asking whether a patient had already been referred and was receiving care for that impairment) based on feedback from the PFAC and external advisory committee. This addition enhances the appropriateness of the system’s recommendation for patients to schedule a visit with their oncology team for further assessment and possible referral to a rehabilitation service. Usability feedback led to design choices that improved navigation, including the addition of definitions accompanying question scales to ensure clarity in responses, and structuring the resource library by impairment and resource type to enable patients to easily locate resources that best match their needs and preferences. Finally, to address privacy and accessibility concerns, REACH was designed to function across multiple devices without storing personal health information locally. These iterative refinements underscore the value of continuous knowledge user engagement during system development.

### Alignment With Best Practices and Recommendations

Our iKT approach used multiple strategies to engage knowledge users, including regular meetings, working groups, surveys, and workshops, to facilitate decision-making for the design of the REACH system for its target populations and clinical settings. These strategies were facilitated through dedicated resources and funding to support these collaborative activities. Our approach is consistent with the findings of a scoping review on iKT approaches in health care, which identified a variety of iKT approaches used such as committees and working groups, meetings and presentations, and consultation and deliberative dialogue [25]. In addition, our iKT approach to developing REACH is consistent with national and international recommendations for the development of ePRO systems for clinical practice. The International Society for Quality of Life Research PRO Implementation Guide and the National Comprehensive Cancer Network Electronic Health Record Oncology Advisory Group outline several considerations for the development of ePRO systems, such as the selection of measures used, frequency of assessments, methods for scoring, interpreting, and displaying assessment results, and approaches to minimize patient and clinician burden and maximize action in response to ePRO completion [26,27]. The Canadian PROs Position Statement recommends that ePRO systems are co-designed with patients and families and that patient data is used in real-time to enhance care and address unmet needs [28]. Finally, priority recommendations developed by Mazariego et al [29] for the implementation of PROs into routine cancer care include the development of strong partnerships with key knowledge users (ie, patients, family members, clinicians, and system developers). Aligned with these recommendations, the development of REACH followed a structured approach to integrate best practices for ePRO system design to ensure the ePROs in REACH are clinically relevant, meaningful, and easy to complete, and that ePRO data is used in real-time to present resources directly to patients to support self-management.

### Limitations and Design Considerations

While our iKT approach ensured that key knowledge users played an active role in shaping the system, there are limitations to this approach that warrant consideration. Despite efforts to incorporate diverse perspectives, it is likely that PFAC members had a greater interest in the use of eHealth interventions for cancer care, which may not fully represent the design perspectives and needs of patients with lower digital or health literacy. In addition, differences in priorities across knowledge user groups may have influenced key design decisions. For instance, while health care providers emphasized minimizing clinical burden and maintaining a patient-driven approach, some patients may benefit from more direct clinical involvement. The PFAC’s motivation and interest, combined with providers’ preference to rely on patients to act on REACH’s prompt to schedule a visit with their oncology team, may limit the system’s feasibility and acceptability for patient subgroups known to have lower engagement with ePRO systems. Similarly, although psychosocial concerns such as distress, anxiety, and fear of recurrence are highly prevalent and overlap with many physical symptoms, these domains were not included in the initial version of REACH. This decision reflected an intentional effort to maintain a narrow scope during the system’s first iteration, focused on persistent physical impairments, in order to keep both the system development and implementation process feasible. In addition, some participating centers had existing distress screening processes in place. However, this design choice may limit the system’s responsiveness to broader patient needs. We are currently assessing the feasibility and acceptability of REACH when implemented into routine care at 4 centers in Canada, and the results of the evaluation will inform refinements to the system, including the potential expansion of symptom domains.

### Conclusions

iKT can serve as a model for developing ePRO-based systems, ensuring they are user-friendly, clinically relevant, and effectively integrated into routine cancer care. This paper describes the use of an iKT approach to develop an ePSM called REACH. By integrating best practices for ePRO system design and actively engaging key knowledge users throughout development, REACH was designed to include ePROs that are clinically relevant, meaningful, and easy to complete, and supported by features that enhance system navigation and patient self-management by linking patients to tailored resources in real-time. REACH has been implemented into routine cancer care at four Canadian centers for 4 disease sites (breast, colorectal, lymphoma, and head and neck), and its implementation is being evaluated to inform system refinements to support patient uptake, feasibility, and acceptability [30].

## Supplementary material

10.2196/74123 Multimedia Appendix 1 Demonstration of user-profile creation on REACH.

10.2196/74123 Multimedia Appendix 2 Demonstration of assessment completion and resource library navigation on REACH.

## Abbreviations

CanRehab: Canadian Cancer Rehabilitation
ePRO: electronic patient-reported outcome
ePSM: electronic prospective surveillance model
HHF: Healthcare Human Factors
iKT: integrated knowledge translation
ML: machine learning
PFAC: patient and family advisory committee
